# A new journal for progress in Laboratory Medicine

**DOI:** 10.1515/almed-2019-0038

**Published:** 2020-03-20

**Authors:** Imma Caballé

**Affiliations:** President of the Spanish Society of Laboratory Medicine, Barcelona, Spain

Access to appropriate resources is crucial to the successful dissemination of scientific knowledge. Publishing research results is also essential, as it enables the sharing of findings that are significant to the scientific community and society.

The list of publications presented in a curriculum vitae provides an objective measure of academic progress at individual level, facilitates access to funding, and brings a competitive advantage in the evaluation of researchers, universities, and scientific institutions.

The launching of the new journal of the Spanish Society of Laboratory Medicine (SEQC^ML^) contributes to the dissemination of scientific knowledge and will help researchers demonstrate their professional development. Fully aware of the impact that a new journal would have on the career of researchers in the field of Laboratory Medicine in Spain led us to include it in SEQC^MC^ 2018–2020 Strategic Plan.

The members of our Society, and more specifically the components of our Scientific Board, have long advocated the creation of an indexed journal that is also published in English. The Board of SEQC^ML^ decided to engage in an ambitious project that will be key to the dissemination of scientific knowledge in our field.

In 2020, our Society is launching an online, open-access, bilingual (English–Spanish) journal aimed at improving the visibility of the scientific literature on Laboratory Medicine.

The new journal will enhance the flow of original articles and scientific documents on our field with their publication in a high-impact, open-access journal.

The number of publications of a researcher throughout their academic career (or in a specific period of their career), the impact of the journal where they were published, and the number of citations received are some of the criteria used to evaluate their scientific activity. The use of these “bibliographic indexes” has largely contributed to expand the reach of scientific publications.

The use of impact factor as an indicator, however, has been a subject of controversy [1], [2], although advances have been made in its design and use, and other options have been explored [3]. Other impact factors used apart from the Journal of Citation Reports include SCImago and Google Scholar. This new journal is intended to play a relevant role in impact factors, as it will provide evidence of the results obtained. An experienced, highly-qualified Board of Editors has been established to decisively contribute to making our vision a reality. Thus, we invite the scientific community to submit their papers.

English has become the global language of communication in the field of scientific research. However, it is essential that knowledge reaches an audience for whom English is a barrier. For this reason, the Board of Editors decided to create a bilingual journal that was published both, in English and Spanish. Publishing in two languages will help laboratory researchers and professionals improve the impact of their scientific works.

The availability of high-quality online bibliographic databases led us to choose an open-access journal. Providing open access will help the journal to achieve a high impact factor.

The name of the journal, *Advancements in Laboratory Medicine*, expresses our aim that scientific findings are applied to Laboratory Medicine practice.

The ultimate role of Laboratory Medicine is to provide data that facilitates the detection or changes the course of a disease, thereby improving the health of the population. We are firmly committed to this approach, and with this purpose this journal is born.

This is an exciting project that requires the involvement and collaboration of the scientific community to create a high-quality journal that facilitates the improvement in scientific knowledge on Laboratory Medicine.
